# Modulation of Mammary Gland Development and Milk Production by Growth Hormone Expression in GH Transgenic Goats

**DOI:** 10.3389/fphys.2016.00278

**Published:** 2016-06-29

**Authors:** Zekun Bao, Jian Lin, Lulu Ye, Qiang Zhang, Jianquan Chen, Qian Yang, Qinghua Yu

**Affiliations:** ^1^College of Veterinary Medicine, Nanjing Agricultural UniversityNanjing, China; ^2^Shanghai Transgenic Research CenterShanghai, China

**Keywords:** mammary gland, growth hormone, development, IGF-1, milk production

## Abstract

Mammary gland development during puberty and reconstruction during pregnancy and lactation is under the control of circulating endocrine hormones, such as growth hormone, which are released from the pituitary. In this study, we explored the influence of overexpression of growth hormone in the mammary gland on breast development and milk production in goats. Using transcriptome sequencing, we found that the number of highly expressed genes was greater in *GH* transgenic goats than non-transgenic goats. Furthermore, KEGG pathway analysis showed that the majority of the genes belonged to the MAPK signaling pathway and the ECM-receptor interaction pathway. The expression of genes related to breast development was further confirmed using qRT-PCR. Interestingly, both milk production and milk quality were increased. The results of these experiments imply that overexpression of growth hormone in the breast may stimulate breast development and enhances milk production by modulating alveolar cell proliferation or branching through the MAPK signaling pathway.

## Introduction

Physiologically, mammary glands undergo significant changes during lactation to prepare for milk production, which is controlled by hormones secreted by the endocrine system (Watson and Khaled, 2008). The serum concentration of prolactin (PRL) and GH are significantly increased, which activates PRLRs and growth hormone receptors (GHRs) to influence ductal and alveolar development and differentiation. Therefore, GH plays a critical role in mammary gland development and milk production (Shingu et al., 2004). GH can not only increase milk protein synthesis, but can also stimulate mammary gland epithelial cell differentiation and proliferation (Macias and Hinck, 2012).

Mammary glands are responsible for milk production and delivery to newborns, which is their only food source. Therefore, mammary gland growth during lactation is important for the development of newborns. Additionally, the main product of dairy animals is milk; therefore, it is necessary to determine how GH improves milk production. Mammary gland development occurs in three distinct and differentially regulated stages: embryonic, pubertal and adult. The mammary glands maintain their ability to produce milk during pregnancy, lactation and involution. In this study, we focused on changes in gene expression following GH overexpression during lactation.

Goat milk is believed to be more easily digestible and less allergenic than cow's milk. The goat milk is also high in calcium and fatty acids but low in cholesterol, which makes it an ideal food especially for the old, weak, sick and disabled (Strzalkowska et al., 2012; Gantner et al., 2015). However, the low yield of goat milk restricts it widely use. In our previous study, we produced a transgenic goat model that overexpressed *GH* in the mammary glands and verified increased *GH* expression in the breast (Zhang et al., 2014). The aim of this study was to determine the effect and mechanism of *GH* overexpression in the breast on breast development and milk production.

## Materials and methods

### Ethics statement

This study was approved by the Ethical Committee of Animal Experiments of the College of Veterinary Medicine at Nanjing Agricultural University. All animal care and use procedures were conducted in strict accordance with the Animal Research Committee guidelines of the College of Veterinary Medicine at Nanjing Agricultural University.

### Experimental animals and tissue collection

Five-year-old *GH* transgenic (TG) and non-transgenic (NG) Saanen dairy goats were raised on the farm at the transgenic research center in Shanghai, China. All goats were healthy and fed with the same fodder. During housing, the health status of all animals was monitored twice a day. No adverse events were observed. We obtained six mammary gland tissue samples from Saanen GH transgenic goats (*n* = 3) and non-transgenic goats (*n* = 3). The tissues were collected during the goats lactation. The mammary tissue samples were harvested and immediately frozen in liquid nitrogen and preserved at −80°C.

Milk samples were collected from TG and NG Saanen dairy goats on the 1st, 7th, 14th, and 30th day of lactation, and milk production, lactose content, milk fat, protein content, density, milk solids, non-fat milk solids and conductivity were detected.

### RNA isolation and transcriptome sequencing

Total RNA was extracted from the mammary gland samples of six Saanen goats using TRIzol reagent. The quantity and purity of the isolated RNA was analyzed prior to the generation of sequencing libraries using the Agilent 2200 TapeStation Systems (Agilent, USA). Sequencing was performed using the Illumina HiSeq 2500 sequencing system. The goat reference genome sequences were downloaded from NCBI. After removing the low quality reads and clipping the adapter sequences, clean reads from each sample were aligned to the goat reference genome using TopHat (Trapnell et al., 2009), which is a gapped aligner that is capable of discovering splice junctions *ab initio*. TopHat reports aligned results from the two mapping steps in shoot apical meristem format for further analysis. Aligned reads from TopHat mapping were subjected to Cufflinks (Trapnell et al., 2010). Gene expression was estimated using Cufflinks and normalized by calculating the reads per kilo base per million mapped reads (RPKM) for each gene and was annotated with NCBI genome assembly (Mortazavi et al., 2008).

### KEGG pathway analysis

To gain an overview of gene pathway networks, KEGG (Kyoto Encyclopedia of Genes and Genomes) analysis was performed using the online KEGG automatic annotation server (https://david.ncifcrf.gov/summary.jsp).

### Confirmation of RNA sequencing results

According to related research and the RNA-seq results, 15 representative genes were verified using qRT-PCR. The assays were performed in samples following RNA preparation. Total RNA was extracted and assessed in RNA sequencing experiments, and DNA was digested using DNase. First strand cDNA was synthesized using a PrimeScript™ RT Reagent Kit with gDNA Eraser (TaKaRa, China) according to the manufacturer's instructions. Real-time PCR was conducted using the 7500 Real-Time PCR System (ABI, USA) and EvaGreen qPCR MasterMix-Low Rox (Abm, Canada). PCR amplification was conducting using the following conditions: 95°C for 10 min, followed by 35 cycles of 95°C for 30 s and 60°C for 34 s. Each sample and negative controls (no template) were run in triplicate. The gene-specific primers are listed in Table 1. The transcriptome sequencing data of the 15 identified genes is shown in the Supplementary Material.

**Table 1:** **QRT-PCR primers used to evaluate the RNA-seq data**.

**Primer names**	**Type**	**Sequences**	**Product length**
GHR-F	Forward	CATAGTGCGGTCTGCTTCCA	250 bp
GHR-R	Reverse	GGTGTGGCTTCACTCCCAG	
IGF1R-F	Forward	GTGGTGACAGGCTACGTGAA	186 bp
IGF1R-R	Reverse	CCCAGCTTTGATGGTCAGGT	
ACTB-F	Forward	CACCACACCTTCTACAA	106 bp
ACTB-R	Reverse	TCTGGGTCATCTTCTCAC	
IGF1-F	Forward	GGATGCTCTCCAGTTCGTGT	161 bp
IGF1-R	Reverse	TGAGAGGCGCACAGTACATC	
IGF2-F	Forward	CAACAGCTGACCTCATTTCCC	243 bp
IGF2-R	Reverse	TGCGTATTGCAAACCGAACAG	
IGFBP3-F	Forward	TTCCTGAACACACTCAGCCC	212 bp
IGFBP3-R	Reverse	GTGGCCACAGTCTACTTGCT	
MST1R-F	Forward	CCGATGTGTGGTCGTATGGT	213 bp
MST1R-R	Reverse	CTGCTAGCTCTCCGAAGGTG	
TGFA-F	Forward	CGCGCTGGGTATTTTCTTGG	129 bp
TGFA-R	Reverse	AACTGACTGTGGGAAGCTGG	
EGFR-F	Forward	TCCTTCACACGTACTGCACC	172 bp
EGFR-R	Reverse	AAAACTGGCCGTGTTGCTTC	
FGF7-F	Forward	AAGTTGCACAGGGCAGACAA	83 bp
FGF7-R	Reverse	GCTGTTTGCTACTTGACTTTTGTTC	
FGFR2-F	Forward	AAGCTGACCAAGCGTATCCC	207 bp
FGFR2-R	Reverse	TTTGCCCAGCGTCAGCTTAT	
TGFB1-F	Forward	AACCTGTGTTGCTCTCTCGG	110 bp
TGFB1-R	Reverse	GAGGTAGCGCCAGGAATTGT	
WNT5A-F	Forward	CACTGGTTCACCCTGTAGCC	189 bp
WNT5A-R	Reverse	TCTCTAGGCACAGCCTCTCA	
COL4A5-F	Forward	CGGGACTTCCAGGACTTTCT	230 bp
COL4A5-R	Reverse	GTGGACCTTGAAAACCTGTAGGA	
COL6A1-F	Forward	ATCGGGCCAAAAGGATACCG	119 bp
COL6A1-R	Reverse	TCTTCACCCCTCTCACCCAT	
COL6A5-F	Forward	GCATTTGCATCCTGGCTCTG	176 bp
COL6A5-R	Reverse	AAAGCAGTTGGTTTTCCCGC	

### Morphology observation of mammary gland

The mammary gland was fixed in Bouin's fluid for 48 h at room temperature. After fixation, samples were cut consecutively into five blocks by using a scalpel, in parallel with the vertical section and cross-section. The blocks were dehydrated and then embedded in paraffin. Six micrometer sections were cut from the paraffin block using a rotary microtome. All sections were mounted on slides and stained with hematoxylin-eosin (HE).

### Statistical analysis

All statistical analyses were performed by using SPSS 17.0. The statistical analysis for gene analysis and milk composition analysis was performed using independent *t*-test. The comparison of the means was performed, and the differences were considered significant if *P* < 0.05

## Results

### Global analysis of gene expression in the mammary glands of NG and TG

To assess the mammary gland transcriptome in GH transgenic goats, we constructed cDNA libraries for the different groups. After filtering, the sequences of the six libraries were mapped to the goat reference genome and the number of mapped genes was complied with the requirements. The RAW data were submitted to NCBI as SRA, and the accession numbers were SRR3659132 and SRR3659145 (https://www.ncbi.nlm.nih.gov/sra/?term=SRR3659132). We observed that 85.64 and 81.10% of the genes mapped uniquely to only one location and could be assigned to a single annotated goat gene in the NG and TG libraries, respectively. We evaluated the expression of genes using RPKM based on methods described by Mortazavi (Mortazavi et al., 2008). To further assess the transcriptome data, the RPKM values were divided into five groups (Table 2): RPKM < 0.1, 0.1 < RPKM < 1, 1 < RPKM < 10, 10 < RPKM < 100, and RPKM > 100. The transcriptome results revealed that the most frequent group in the NG was 1 < RPKM < 10, whereas in the TG, the most frequent group was 10 < RPKM < 100. Moreover, the RPKM > 100 group in the TG was higher compared to that in the NG. We analyzed the genes in the RPKM > 1 group in the following experiments.

**Table 2:** **Distribution of the expression values of the differentially expressed genes**.

**RPKM range**	**NG (frequency)**			**TG (frequency)**		
	**1**	**2**	**3**	**1**	**2**	**3**
(0, 0.1)	5733	5372	5407	4014	3767	3685
(0.1, 1)	3455	3816	3825	2361	2137	2189
(1, 10)	7491	7368	7464	5928	5327	5403
(10, 100)	2494	2608	2474	6493	7421	7446
(100, INF)	205	214	209	584	668	657

### Differentially expressed genes in TG and NG

Genes were determined to be differentially expressed based on the false discovery rate of the adjusted RPKM > 1 based on *P* < 0.01 and fold-change in expression >2. This resulted in the identification of 4451 differentially expressed genes; 2274 were expressed higher and 2217 were expressed lower in the TG.

### KEGG pathway enrichment analysis

**Figure 1:**
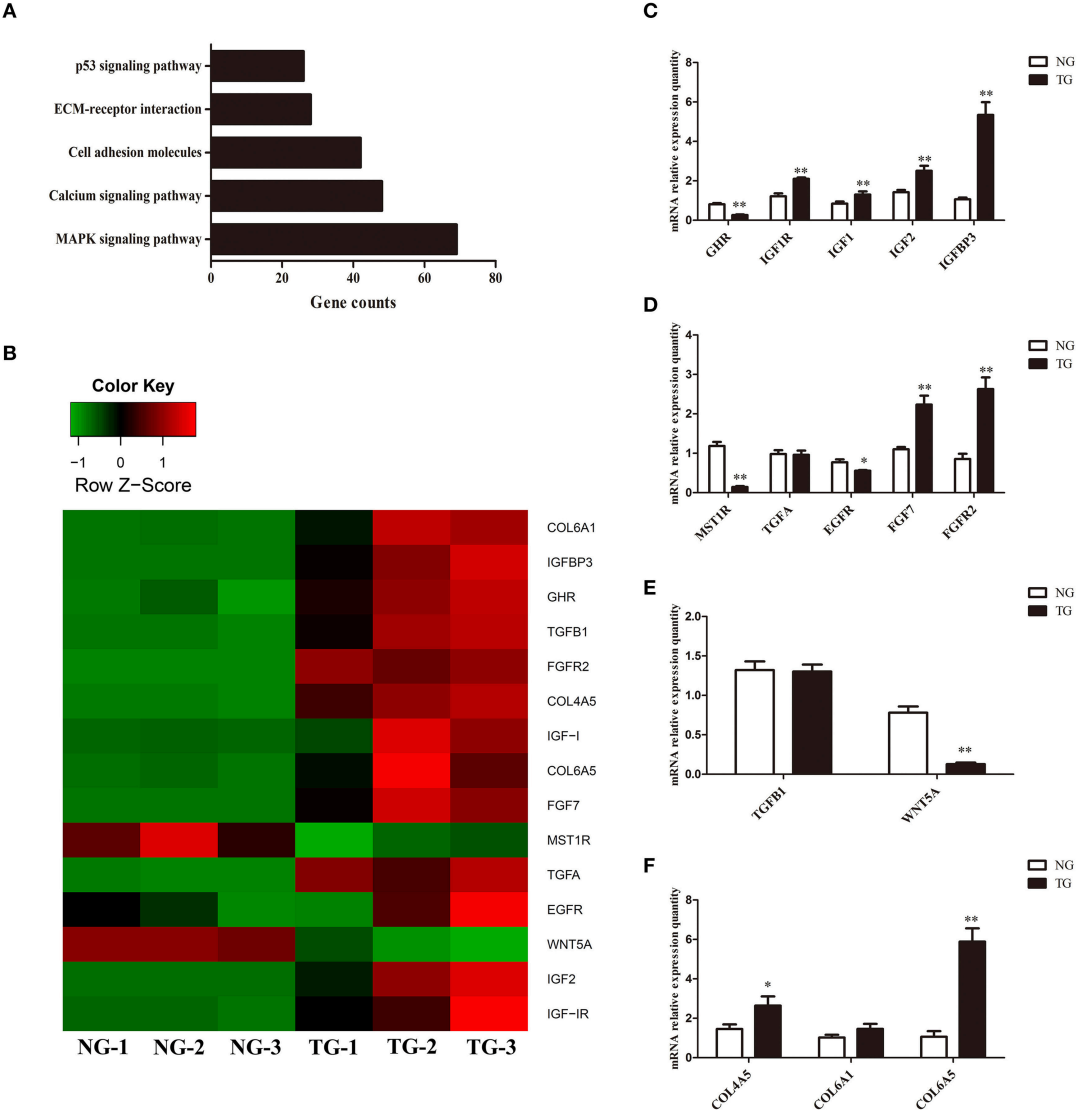
**Gene expression analysis. (A)** KEGG pathway analysis of the RNA sequencing data. The x-axis represents the number of genes for each pathway. **(B)** Heat map of the genes expressed in the TG and NG groups. NG, non-transgenic goat. TG, transgenic goat (*n* = 3 per group). Red represents high expression and green represents low expression. **(C–F)** Differences in gene expression between the NG and TG groups as detected using qRT-PCR. The x-axis represents the name of the detected genes and the y-axis represents the relative mRNA expression for each detected gene. The data are expressed as the mean ± SE (*n* = 3). *P < 0.05; **P < 0.01.

KEGG pathway analysis, which is an alternative approach to categorize gene functions with a focus on biochemical pathways, was performed on the annotated genes. A total of 991 genes were assigned one or more KEGG annotations and were mapped to KEGG pathways. A total of 37 pathways were significantly enriched, including the MAPK signaling pathway, calcium signaling pathway, cell adhesion molecules, ECM-receptor interaction pathway, and p53 signaling pathway (Figure 1A).

### Confirmation of the RNA-seq results using qRT-PCR

To validate our RNA-seq results, we subjected 15 differentially expressed genes to qRT-PCR. The qRT-PCR results largely confirmed our RNA-seq data (Figure 1C). Although the fold-change values in the qRT-PCR and RNA-seq data were not directly related, they displayed similar tendencies (Figure 1B). The expression of eight genes was increased (*IGF1R, IGF1, IGF2, IGFBP3, FGF7, FGFR2, COL4A5* and *COL6A5*) and that of four genes were decreased (*GHR, MST1R, EGFR* and *WNT5A)*. There was no significant change in the expression of *TGFA, TGFB1* and *COL6A1*.

### The effect of GH on mammary gland structure

**Figure 2:**
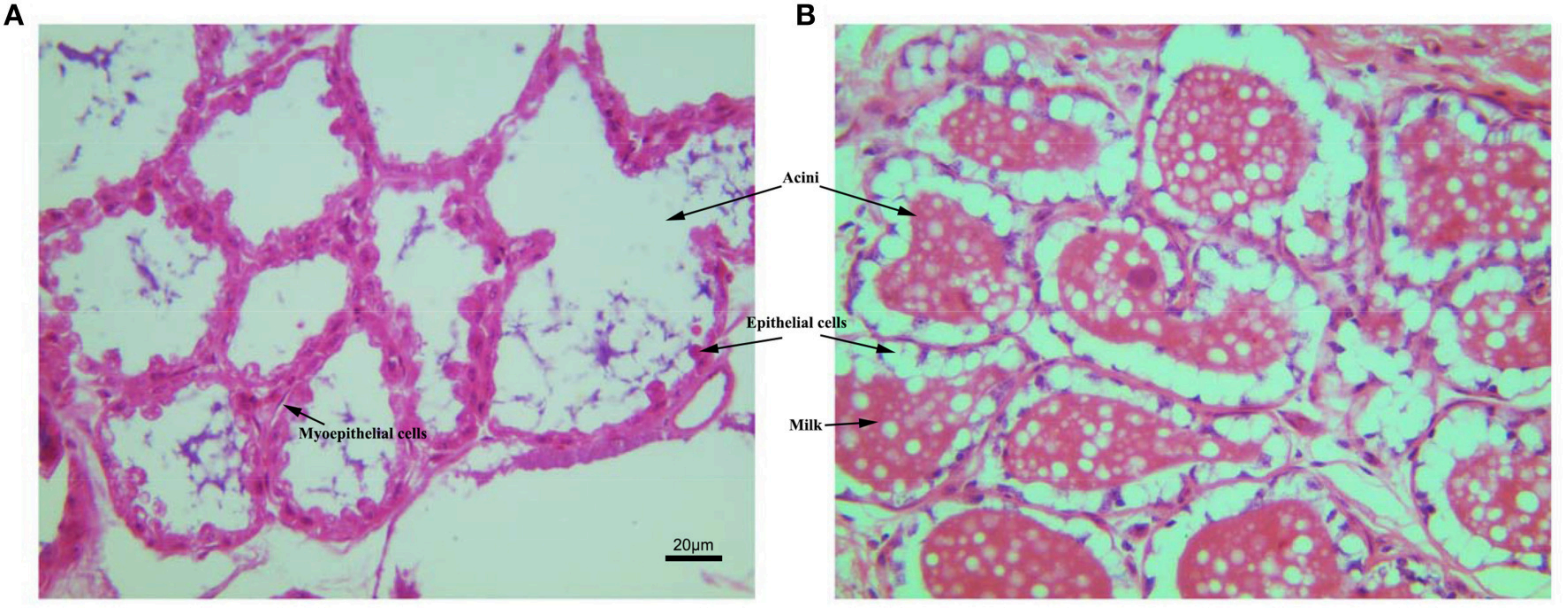
**The histological structure of mammary gland stained with HE**. **(A)** Mammary gland of non-transgenic goat (NG); **(B)** mammary gland of GH transgenic goat (TG). The Mammary glands of NG and TG were collected in the same lactation period, then fixed and cut into paraffin section. The tissues were stained with HE. The milk, aicni and myoepithelial cells underneath the epithelial cells were labeled.

The tissue sections of mammary gland stained with HE were shown in Figure 2. The Mammary glands of NG and TG were collected in the same lactation period. Compared with the NG, more goat milk with clear lipid droplet was visible in the acini of TG. The epithelial cells in the NG were rounded, while the shape of epithelial cells in TG was more flat with abundant milk. The myoepithelial cells underneath the epithelial cells were also detected around the epithelial cells.

### Milk detection

**Figure 3:**
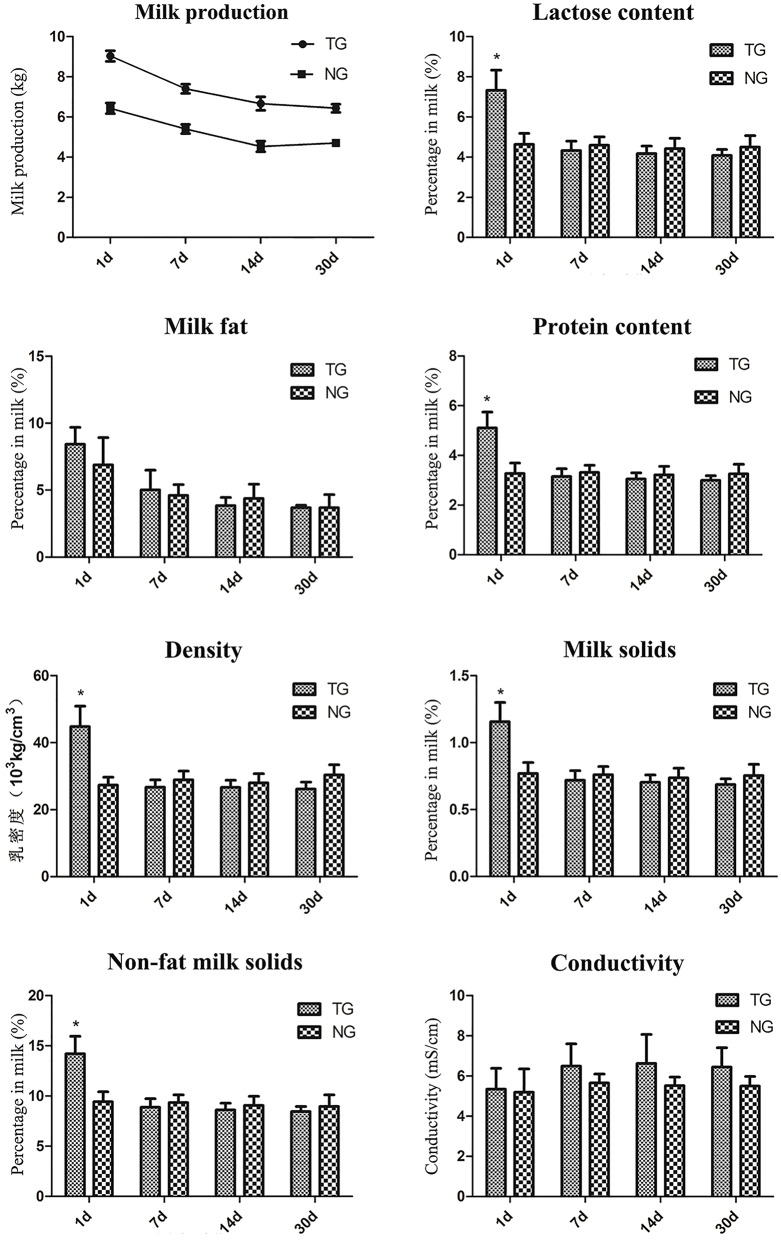
**Milk sample detection**. Eight milk samples were assessed for different measures, including milk production, lactose content, milk fat, protein content, density, milk solids, non-fat milk solids and conductivity. The x-axis represents the date that each sample was collected and the y-axis represents the value of each measurement. *P < 0.05.

Milk production was significantly increased in the TG group compared with the NG group during the lactation detection period (Figure 3). There were no significant changes in milk fat and conductivity in the TG group during the lactation detection period. The lactose content, protein content, density, milk solids and non-fat milk solids were significantly increased in the TG group compared with the NG group on the first day of lactation. However, there were no significant differences in these measures during the remaining three lactation detection time periods.

## Discussion

Mammary gland development is dynamic and divided into three stages: embryonic, pubertal, and adult (Gjorevski and Nelson, 2011). Adult mammary gland growth is an important stage of preparation during pregnancy and lactation in mammals. GH plays a critical role in this stage (Molik et al., 2010; Macias and Hinck, 2012). Higher exogenous overexpression of *GH* was detected in mammary gland in TG goats (Zhang et al., 2014). This allows us to study the effects of GH on mammary gland growth.

Mammary gland development during lactation requires growth factors that increase the proliferation and survival of mammary epithelial cells. Overexpression of *GH* stimulates the expression of growth factors. KEGG pathway analysis showed that these genes were involved in mammary gland physiology, including the MAPK signaling pathway, calcium signaling pathway, cell adhesion molecules, ECM-receptor interaction pathway and p53 signaling pathway. Based on these results, we selected 15 genes and examined differences in the expression levels between the TG and NG.

**Figure 4:**
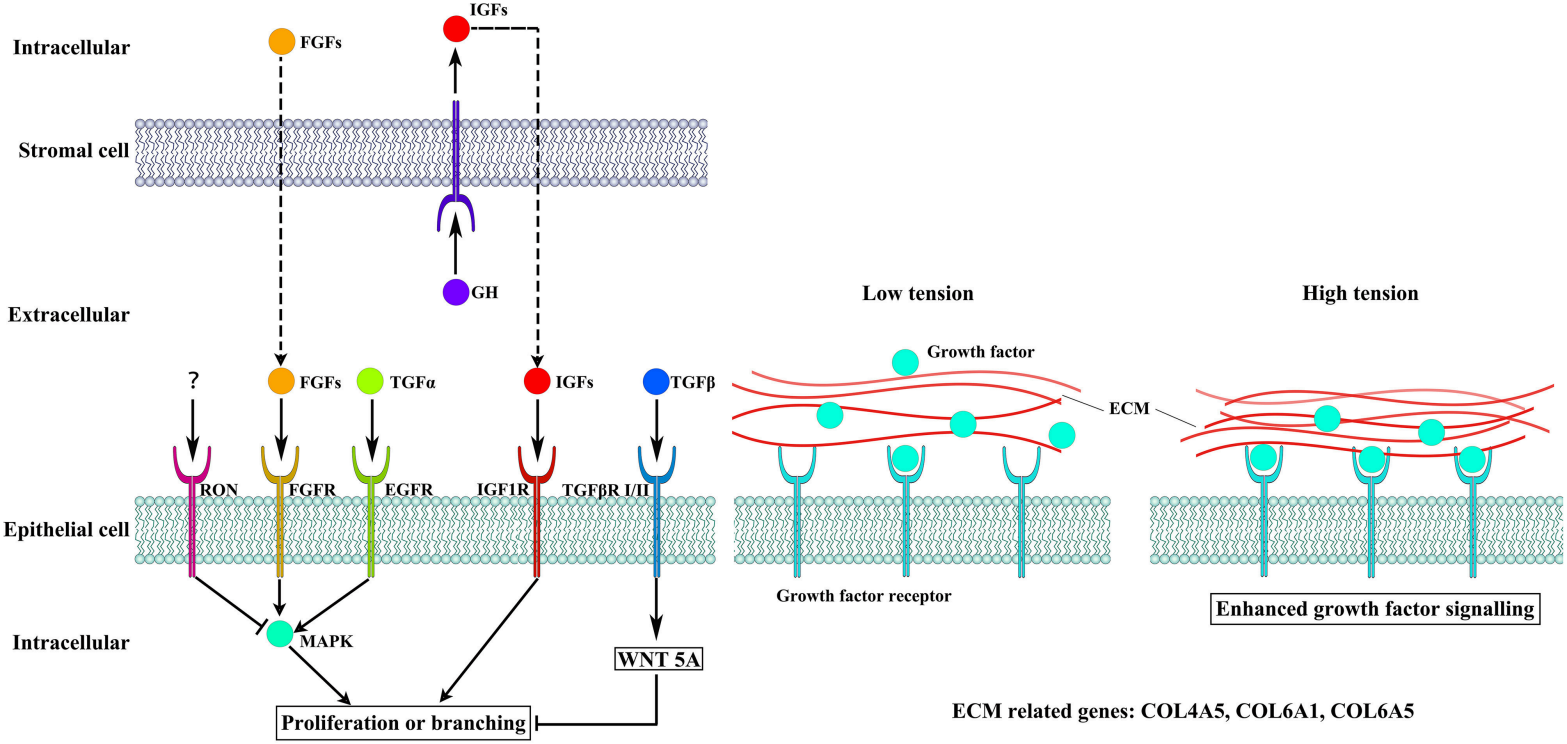
**The different pathways altered by overexpression of GH**. Cellular crosstalk between the epithelial and stromal compartments is mediated by growth factors, including IGF1, EGF, and FGFs, which bind to their receptors to induce cell proliferation and branching. The WNT5A pathway can also influence epithelial cells proliferation. High tension from cell-generated mechanical stress can promote the binding of growth factors to their receptors.

GH binds to GHRs, which activate its effects. Our results showed that overexpression of *GH* altered the expression of *GHR* in the mammary glands. GH induces paracrine signaling by activating GHRs on stromal cells. This process could induce the expression of insulin-like growth factor 1 (IGF1) in stromal cells (Perez et al., 2016). IGF1 binds to cell surface receptors (IGF1R) on epithelial cells (Perez et al., 2016) to stimulate mammary gland epithelial cell growth and development, as well as cellular DNA synthesis (Kleinberg et al., 2000; Perez et al., 2016). Similar to IGF1, insulin-like growth factor 2 (IGF2) also increases cell proliferation and survival by binding to IGF1Rs (Nawathe et al., 2016) (Figure 4). Although in our study expression of *GHR* was decreased, the expression of *IGF1, IGF2* and *IGF1R* was significantly increased in the TG group. Insulin-like growth factor-binding protein 3 (IGFBP3) exerts antiproliferative effects in many cell types by blocking the ability of IGF1 and IGF2 to activate IGF1Rs (Nawathe et al., 2016). Our results showed that *IGFBP3* was significantly increased in the TG group. The upregulation of *IGF1, IGF2*, and *IGF1R* might lead to excessive proliferation of epithelial cells. Increasing *IGFBP3* and decreasing *GHR* might prevent the excessive growth of the mammary glands, which could damage the organism (Figure 1C).

Several receptor tyrosine kinases have profound effects on mammary gland development during puberty, including RON, which negatively regulates mammary gland branching morphogenesis (Meyer et al., 2009). RON is encoded by *MST1R*, which was significantly decreased in our study. The kinetic profile of MAPK activity may determine the final morphogenetic response. Sustained MAPK activation downstream of transforming growth factor-α (TGFα) and epidermal growth factor receptor (EGFR) induces branching. Moreover, transient MAPK activation downstream of fibroblast growth factor 7 (FGF7) and fibroblast growth factor receptor 2 (FGFR2) induces proliferation (Sternlicht et al., 2005; Fata et al., 2007) (Figure 4). Our results show that there was no difference in the expression of *TGFA* between the two groups and *EGFR* was slightly downregulated in the TG group. However, *FGF7* and *FGFR2* were significantly increased in the TG group (Figure 1D). These results suggest that overexpression of *GH* might promote mammary gland proliferation through FGF7 and FGFR2, but does not promote mammary gland branching through TGFα and EGFR. In addition, inhibition of RON might promote mammary gland branching.

Transforming growth factor-β (TGFβ) plays an important role in mammary gland development, and its concentration profile is determined by tissue geometry. In microfabricated gland mammary tissue, branching is inhibited at sites of high concentrations of TGFβ (Nelson et al., 2006). Overexpression of TGFβ1 leads to inhibition of mammary gland development *in vivo* (Pierce et al., 1993), whereas TGFβ-deficient mice exhibit accelerated ductal proliferation and lateral branching (Ewan et al., 2002; Crowley et al., 2005). WNT5A acts downstream of TGFβ *in vivo* (Roarty and Serra, 2007) and is required for TGFβ-mediated inhibition of mammary branching morphogenesis (Roarty and Serra, 2007; Macias et al., 2011; Kumawat and Gosens, 2016) (Figure 4). We detected the expression of *TGFB1* and *WNT5A* in the two groups and found that the expression of *WNT5A* was significantly downregulated in the TG group, whereas there was no change in the expression of *TGFB1* between the TG and NG groups (Figure 1E). These results suggest that overexpression of *GH* might ameliorate the inhibition of mammary development by reducing *WNT5A* expression and not *TGFB1* expression.

Cell-generated mechanical stress can remodel the surrounding matrix and release extracellular matrix (ECM)-bound regulatory molecules, such as growth factors (Vogel and Sheetz, 2006). The binding of these growth factors to their receptors under high tension conditions results in increased growth factor signaling (Sternlicht et al., 2006; Muschler and Streuli, 2010). Cryptic binding sites in the ECM may be unable to access their receptors under low tension conditions; however, under high tension, the ECM may expose these binding sites, which allows them to engage more integrin receptors and trigger enhanced downstream signaling (Vogel and Sheetz, 2006; Insua-Rodriguez and Oskarsson, 2016) (Figure 4). Collagen IV is an important component of the ECM in the mammary glands (Siu and Cheng, 2008). Therefore, we detected three genes related to the expression of collagen IV, including *COL4A5, COL6A1*, and *COL6A5* (Khoshnoodi et al., 2008; Meuwissen et al., 2015). Two of these genes were significantly upregulated, which suggests that overexpression of GH in the mammary glands enhances mechanical stress and promotes growth factor activation (Figure 1F).

Goats' milk is highly nutritious, contains essential vitamins and minerals and is an ideal food for the whole family to enjoy. However, limited goat milk yield makes it expensive and not easy to get. To determine whether overexpression of *GH* could influence milk composition during mammary gland growth, we assessed several indicators of milk composition. Our results showed that milk production was significantly increased during the lactation period. This result was also supported by the mammary gland development observed by the HE staining. We found that more goat milk was visible in the acini of TG and the shape of epithelial cells in TG was more flat with abundant milk, which is inconsistent with the high milk yield in TG. There were no changes in milk fat or conductivity during our detection period. Moreover, the lactose content, protein content, density, milk solids and non-fat milk solids were increased on the first day of lactation. However, similar levels of these measures were observed between the two groups in the subsequent detection time periods. These results indicate that overexpression of *GH* increase milk production, but do not change the composition of milk. This phenomenon will be promising for the goat milk consumption.

Overall, our results showed that overexpression of *GH* stimulate mammary gland development. This process involves the upregulation of several growth factors, which activate mammary gland proliferation and branching. In addition, inhibition of mammary gland development was decreased. Moreover, mechanical stress was increased, which could improve the function of growth factors. Although overexpression of *GH* might alter mammary gland development, the milk composition remains stable.

## Author contributions

ZB and JL: substantially contributed to the conception and implementation of the experiment; JC and QZ: raised the goats; ZB and LY: performed sample collection and analysis; QY and QY: contributed to the concept, design of the work and financial support.

### Conflict of interest statement

The authors declare that the research was conducted in the absence of any commercial or financial relationships that could be construed as a potential conflict of interest.
