# Supplementary material for: Modulation of Mammary Gland Development and Milk Production by Growth Hormone Expression in GH Transgenic Goats
Source: Front Physiol. 2016 Jun 29;7:278. doi: 10.3389/fphys.2016.00278 (PMC4926316; doi:10.3389/fphys.2016.00278)
Supplement: Supplementary file 2 [file DataSheet1.PDF]

Supplementary information captions.

Fig S1. Gene expression of downstream of MAPK

Expression of the genes in NG and TG detected by qRT-PCR, x-axis is the symbol of the detected genes and y-axis is the mRNA relative expression quantity of each detected gene. FOS, MYC and JUN are the downstream of the MAPK pathway, which relate to cell proliferation and differentiation. Our results showed FOS and JUN were increased significantly, which suggested that the enhancement of FGF and FGFR expression had activated MAPK pathway. This result confirmed the conclusion of our manuscript. Data are expressed as mean ± SD (n=3). **P < 0.01.

Table S1. Data of RNA sequencing.

The data of RNA sequencing was submitted as supplementary material, each of NG and TG had three replicates with the sequencing. The data included gene length, gene reads and gene RPKM.
