# Supplementary figures and images for: Modulation of Mammary Gland Development and Milk Production by Growth Hormone Expression in GH Transgenic Goats
Source: Front Physiol. 2016 Jun 29;7:278. doi: 10.3389/fphys.2016.00278 (PMC4926316; doi:10.3389/fphys.2016.00278)

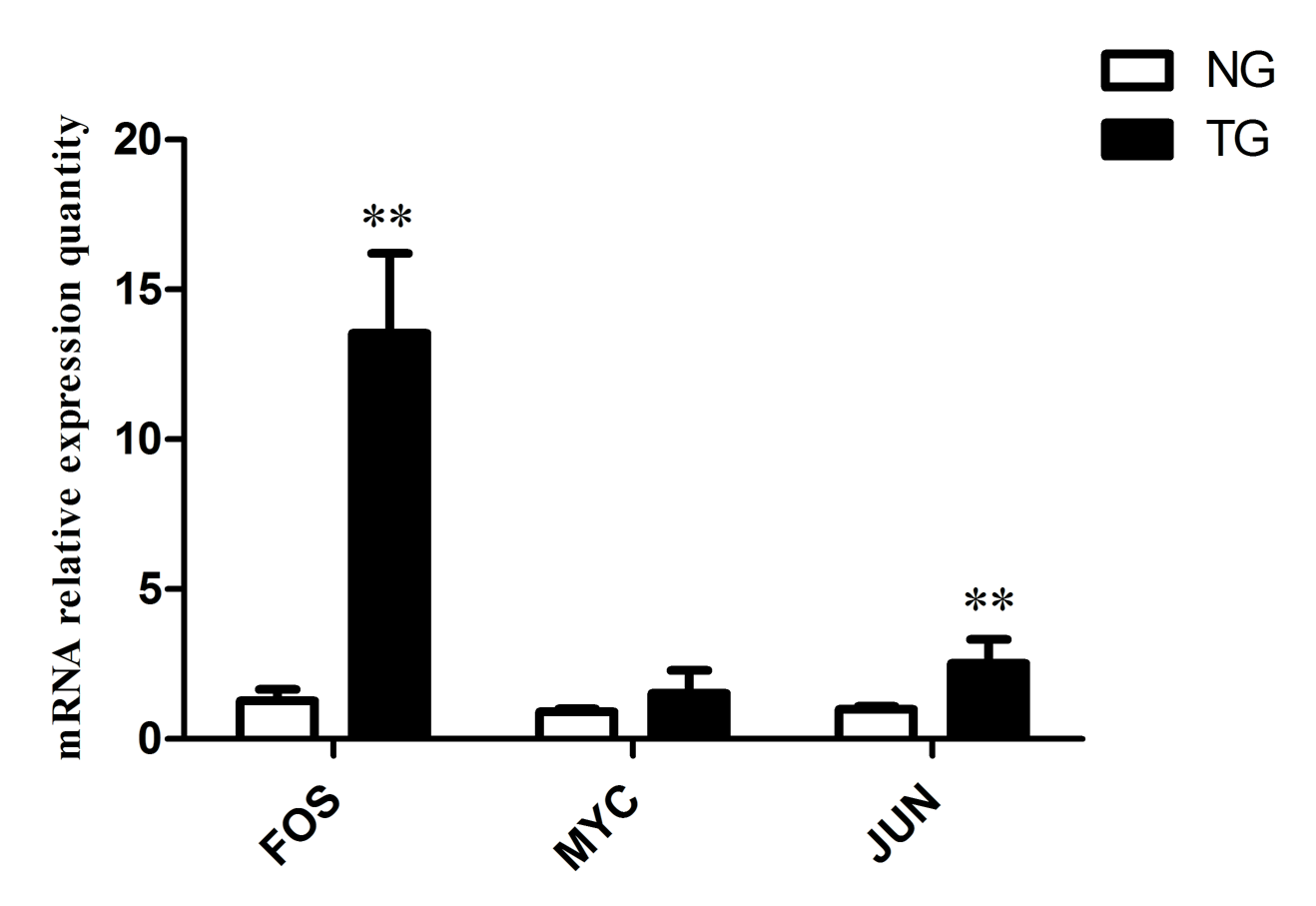

Supplement: Supplementary file 3 [file Image1.TIF]
